# Relationship between self-reported and objectively measured manual ability varies during the first year post-stroke

**DOI:** 10.1038/s41598-020-61834-1

**Published:** 2020-03-20

**Authors:** Netha Hussain, Margit Alt Murphy, Åsa Lundgren-Nilsson, Katharina S. Sunnerhagen

**Affiliations:** Institute of Neuroscience and Physiology, Sahlgrenska Academy, University of Gothenburg, Gothenburg, Sweden

**Keywords:** Neurology, Neurological disorders, Stroke

## Abstract

Self-reported outcomes provide unique insights about an individual’s perceived manual ability after stroke. This study aimed at determining how the relationship between objective kinematic variables obtained from the target-to-target pointing task and self-reported manual ability varies during the first year in individuals after stroke. Sixty-six individuals from the Stroke Arm Longitudinal study at the University of Gothenburg (SALGOT) cohort were assessed using ABILHAND questionnaire and kinematic analysis at five timepoints between the 10^th^ day and 12^th^ month after stroke. Kinematic analysis was performed using a target-to-target pointing task in a virtual environment. Spearman’s correlation was used to determine the extent of correlation between ABILHAND logits and kinematic variables. The correlations varied with time within the first year after stroke. The correlations were low or very low early after stroke and became moderate to high after 6 months for objective measures of movement time and smoothness, but remained low to moderate for mean velocity and low for peak velocity. Due to this discrepancy between self-perceived and objective assessments of arm function, a combination of self-reported and objective assessments of upper limb should be used as outcome measures, especially in the acute and subacute stages after stroke.

## Introduction

Self-reported outcome measures could give insights about an individual’s perceived functional ability, especially in performing daily-life tasks outside the hospital setting^1,2^. They provide information about the individual’s difficulties in everyday life, which are not directly observable or quantifiable by others^2,3^. On the other hand, standardized observational scales assess movements by direct observation while objective measurements such as kinematic analysis assess movements by measuring actual movement output using technology. Objective measures are minimally influenced by the observer’s bias and experience, and are often sensitive in measuring subtle, yet important changes^4^. Both self-reported outcome measures and objective measures such as kinematics are recommended to be included as an outcome measure in upper limb stroke rehabilitation trials^5^. However, there is limited knowledge regarding the relationships between objective measures and self-reported measures of manual ability during the recovery process after stroke.

In stroke rehabilitation, re-gaining the independence in daily activities is considered an important therapeutic outcome. In order to re-gain independence, it is important that the individual perceives oneself as having good functional ability^6^. Arm function can be perceived as limited, even with good observed function of the more-affected limb in the subacute and chronic stages of stroke^2,7,8^. About 20–40% of individuals with full or nearly full observed hand function reported decreased self-perceived hand function at the chronic stage of stroke^7,9^. Such a high prevalence of mismatch between observed and perceived function warrants the need for more investigation into this phenomenon.

Kinematic analysis using virtual reality (VR) systems with haptic device provides objective data of post-stroke upper limb function^10^. Kinematic variables obtained from a virtual reality setup can discriminate between levels of impairment in stroke and healthy controls^11^. In addition, VR-based devices are more readily available in clinical settings as evaluation and/or training devices for upper limb^12^. Despite the wide usage of VR-based equipment, the relationship between VR-based kinematic variables of the upper limb and self-perceived manual ability has not been studied in detail.

In individuals with stroke, self-reported manual ability can be assessed using questionnaire-based scales such as ABILHAND^13^. The ABILHAND scale is a unidimensional continuous scale for evaluating the individual’s self-reported difficulty in performing daily bimanual tasks^13^. The relationship between ABILHAND scores and upper extremity outcome measures has been explored in a few studies^14,15^. Manual dexterity assessed using an observational clinical scale explained 39% of the variance in ABILHAND in chronic stage after stroke^15^. Low correlation (0.3–0.4) was reported between objective kinematic measures from the drinking task and ABILHAND logits in a sub-acute stroke population^14^. However, the relationship between self-perceived and objective assessment of upper limb function has not been reported yet for the acute stage of stroke. Furthermore, the knowledge regarding the variation in the relationship between self-reported and objectively assessed arm function during the acute, subacute and chronic stage is limited. Hypothetically, it is likely that correlations change over time during the first year after stroke and that the correlation would become stronger at the later time points when the changes in function reach a plateau and the understanding of one’s own manual ability consolidates. This information might be useful for clinicians to better understand the interaction between the perceived manual ability and objectively assessed arm function during the recovery process. This would allow clinicians to better guide the patients in setting individualized, achievable and realistic goals at all stages after stroke.

The aim of this study was to determine how the relationship between objective kinematic variables obtained from the target-to-target pointing task and self-reported manual ability varies during the first year in individuals after stroke.

## Methods

### Study design

**Figure 1:**
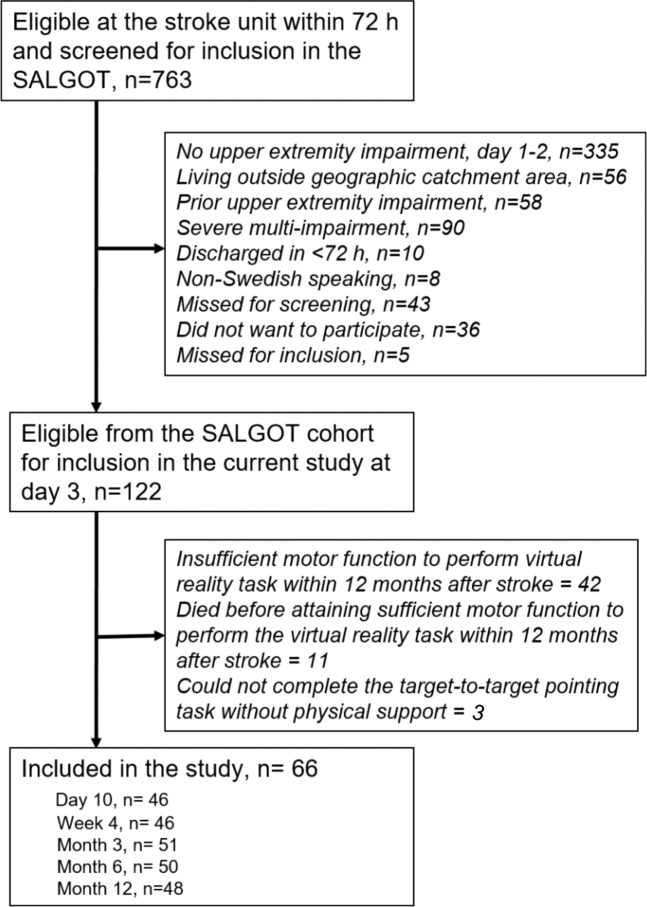
Flowchart of the inclusion process.

The participants of this study were extracted from the Stroke Arm Longitudinal Study at Gothenburg University (SALGOT) cohort of 122 unselected individuals within the first year after stroke^16^. The SALGOT cohort comprised of adults living in Gothenburg urban area with impaired upper limb function (<66 points on Fugl Meyer Assessment of Upper Extremity at 3 days post stroke) following first-ever stroke and admitted within three days of stroke onset. World Health Organization collaborative study criteria was used for determining the diagnosis of stroke^17^. The following were the exclusion criteria for SALGOT: upper limb condition prior to stroke that limits the functional use of the arm, severe multi-impairment or diminished physical condition before the stroke that will affect arm function, life expectancy less than 12 months due to other illness or severity of stroke injury and not Swedish speaking prior to the stroke incident. In total, 66 participants were included in the study and the inclusion process is shown in Fig. 1.

Each individual was assessed five times during the first year after stroke using a battery of clinical and kinematic assessments. The assessments were carried out within 10 days (mean 9.54 days), at 4 weeks, 3 months, 6 months and 12 months after the stroke onset. Day 10, week 4 and month 3 were considered as early subacute stage after stroke, month 6 as late subacute stage and month 12 as chronic stage of stroke.

### Equipment and procedure

The equipment used for the study includes a semi-immersive Virtual Reality (VR) workbench, 3D shuttered glasses and a haptic stylus. The VR workbench has 3D display of virtual space on a mirror when observed through stereoscopic shuttered glasses. The infrared transmitter on the workbench sends signals to the shuttered glasses and synchronizes the image sequence on display, giving the participant an illusion of seeing 3D objects.

The PHANTOM® Omni™ haptic stylus captures kinematic data. The haptic stylus can be moved freely in the virtual workspace (160 × 120 × 120 mm), and gives touch sensation when it comes close to a virtual object. In addition to visual feedback (colour change and disappearance), force feedback is also given when the virtual object is pointed at by the participant. Thus, the participant gets an illusion of touching and manipulating virtual objects with the stylus.

The participant was asked to reach and point at the disc shaped target (~3.0° viewing angle) using the tip of the haptic stylus while being seated comfortably on a chair, wearing 3D glasses and looking into the 3D space shown on the display mirror. A new target appeared at another location when the previous one was pointed at and made to disappear. The participant was instructed to perform the pointing task as fast and as accurate as possible. The task, which consisted of 32 targets, ended when the last of all targets disappeared. The participants performed one or two trials before the assessment in order to acquaint themselves with the VR setup.

### Kinematic variables

Kinematic data was gathered from the haptic device during the task and kinematic variables were extracted from this data using custom-made software Curictus™ and MATLAB^®^ software. Four relevant kinematic variables were extracted from the data: movement time, mean velocity, peak velocity and number of velocity peaks. These kinematic variables were chosen since a previous study showed that they were discriminative for individuals with stroke and healthy controls during the target-to-target pointing task^11^.

The time taken to complete one movement segment was defined as movement time. The maximum absolute velocity recorded during each movement segment was taken as peak velocity. The smoothness of movement was measured by counting the number of velocity peaks in a movement segment. In order to define a peak, the velocity profile was first searched for local minima and maxima. When the difference between a minimum and the next maximum exceeded the cut-off limit of 20 mm/s, it was counted as a velocity peak. Additionally, the time between two subsequent peaks had to be at least 150 milliseconds. A movement segment was defined as the entity between the appearance of a target and its disappearance when pointed at by the stylus. The whole task was divided into 31 movement segments. For each participant, all kinematic variables were calculated as the mean of all 31 movement segments for the entire task.

### Clinical assessment

The self-reported manual ability of individuals with stroke was measured by the ABILHAND questionnaire^13^. ABILHAND evaluates the individual’s perceived difficulty in performing daily bimanual tasks and uses Rasch analysis methodology where raw ordinal data is converted into a unidimensional interval scale with scores presented as logits. ABILHAND contains 23 items, where each item is classified as impossible (0 point), difficult (1 point) or easy (2 points) to perform. When the activity had not been attempted, a question mark symbol was recorded. The raw test scores for each of the items were entered into a Rasch analysis online module (www.rehab-scales.org). The ABILHAND logit score and standard error was extracted for each individual. ABILHAND logit score ranges from about −6 to 6 and higher positive scores imply better self-reported manual ability.

The sensorimotor function of the upper limb was assessed by the Fugl-Meyer Assessment of Upper Extremity (FMA-UE)^18^. A maximum score of 66 points in FMA-UE scale indicates unimpaired upper limb function. Barrow Neurological Institute (BNI) pre-screening, with a maximum score of 9 points, was performed to assess level of alertness, basal communication and co-operation^19^.

### Statistical analyses

The statistical data analyses were done using IBM Statistical Package for Social Sciences™ (SPSS) version 24 for Windows. The normality of the variables was tested using Shapiro-Wilk’s test, histograms and Q-Q plots. All variables were found to be approximately normally distributed, except for ABILHAND logits at week 4, month 3, month 6 and month 12. Spearman’s correlation coefficient was used to measure the correlation between ABILHAND logit scores and kinematic variables. The strength of correlation coefficients was interpreted as 0.00–0.25 (very low), 0.26–0.49 (low), 0.50–0.69 (moderate), 0.70–0.89 (high) and 0.90–1.00 (very high)^20^. Since correlations between 20 pairs of variables were tested, Bonferroni correction was performed, following which the p-value required for significance was adjusted to <0.0025.

### Ethical approval and informed consent

The ethical approval was obtained from Regional Ethical Review Board, Gothenburg, Sweden (No. 225-08). Informed, written consent was obtained from all participants prior to their inclusion in the study. All methods were performed in accordance with the relevant guidelines and regulations of the Regional Ethical Review Board. The SALGOT study was listed in clinicaltrials.gov with the trial registration number NCT01115348.

## Results

**Figure 2:**
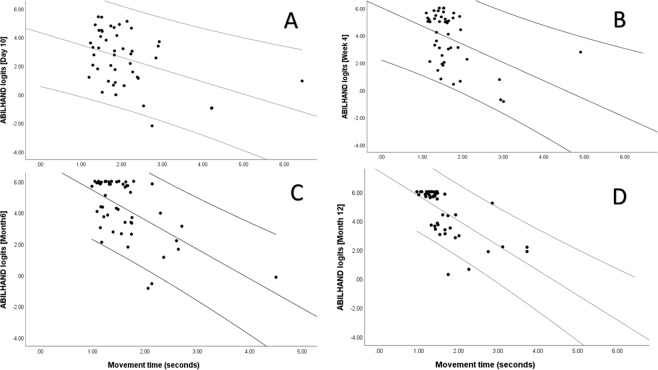
Scatter plot showing the relationship between movement time and ABILHAND logits at (**A**) Day 10, (**B**) week 4, (**C**) month 6 and (**D**) month 12 (clockwise).

The descriptive statistics for demographic data, clinical characteristics, baseline kinematic variables and ABILHAND logits of the study population are shown in Table 1. The correlations between kinematic variables and ABILHAND logits at each time point after stroke are shown in Table 2. The scatter plots showing correlation between the movement time and ABILHAND logits at four different time points are displayed in Fig. 2.

**Table 1:** Demographic data and clinical characteristics of the study population.

Demographic data, clinical characteristics and assessments Mean ± SD, n (%) or median (Q1-Q3)	Overall (n = 66)	Day 10 (n = 46)	Week 4 (n = 46)	Month 3 (n = 51)	Month 6 (n = 50)	Month 12 (n = 48)
Age	65.7 ± 13.4	64.02 ± 13.89	64.62 ± 12.08	64.44 ± 12.81	65.10 ± 13.36	64.67 ± 12.29
Female	27 (41%)	16 (35%)	19 (41%)	22 (43%)	22 (44%)	20 (42%)
Ischemic/Haemorrhagic stroke (%)	81/19	83/17	78/22	74/26	76/24	75/25
Right hand dominant	63 (95%)	44 (96%)	44 (96%)	49 (96%)	48 (96%)	46 (96%)
Right hemiparesis	29 (44%)	20 (44%)	19 (41%)	22 (43%)	22 (43%)	19 (40%)
Inpatient rehabilitation	—	27 (58%)	1 (2%)	none	none	none
FMA-UE score	—	61 (56–64)	64 (60–66)	64 (61–66)	64 (60–66)	64 (61–66)
Score <9 in BNI pre-screening	—	4 (9%)	2 (4%)	4 (8%)	4 (8%)	1 (2%)
ABILHAND logits	—	2.44 ± 1.95	3.84 ± 1.94	4.35 ± 1.69	4.30 ± 1.91	4.66 ± 1.62
ABILHAND standard error	—	1.20 ± 0.49	1.31 ± 0.91	1.20 ± 0.52	1.17 ± 0.53	1.24 ± 0.54
Movement time (s)	—	2.06 ± 0.95	1.70 ± 0.65	1.72 ± 0.78	1.61 ± 0.60	1.62 ± 0.63
Mean velocity (m/s)	—	0.17 ± 0.05	0.19 ± 0.06	0.19 ± 0.06	0.19 ± 0.06	0.20 ± 0.07
Peak velocity (m/s)	—	0.41 ± 0.12	0.42 ± 0.12	0.43 ± 0.13	0.43 ± 0.12	0.44 ± 0.14
Number of velocity peaks (n)	—	3.99 ± 1.72	3.43 ± 1.54	3.41 ± 1.25	3.24 ± 0.97	3.33 ± 1.07

Abbreviations: SD, Standard deviation; Q1–Q3, Quartiles; FMA, Fugl-Meyer Assessment of Sensorimotor Function, ARAT, Action Research Arm Test, BNI, Barrow Neurological Institute.

**Table 2:** Spearman’s correlation analysis between ABILHAND logits and kinematic variables.

	Correlation coefficient of ABILHAND logits (p-value)				
Kinematic variables	Day 10 (n = 46)	Week 4 (n = 46)	Month 3 (n = 51)	Month 6 (n = 50)	Month 12 (n = 48)
Movement time	−0.370 (0.011)	−0.316 (0.031)	−**0.463 (0.001)**	−**0.489 (<0.001)**	−**0.750 (<0.001)**
Mean velocity	0.016 (0.916)	0.157 (0.293)	0.382 (0.006)	0.348 (0.013)	**0.535 (<0.001)**
Peak velocity	−0.141 (0.350)	0.068 (0.651)	0.310 (0.027)	0.154 (0.285)	0.366 (0.010)
Number of velocity peaks	−**0.449 (0.002)**	−0.302 (0.039)	−0.371 (0.007)	−**0.538 (<0.001)**	−**0.659 (<0.001)**

Correlation coefficients with p < 0.002 marked in bold letters.

**Figure 3:**
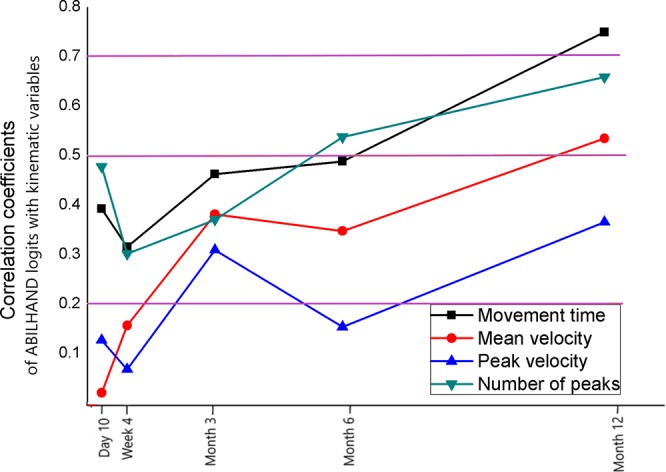
Line graph showing the correlation coefficients between ABILHAND logits and kinematic variables between day 10 and month 12 after stroke. The three horizontal lines from top to bottom mark the limits for high (0.7), moderate (0.5) and low (0.2) correlation respectively.

The correlations between kinematic variables and ABILHAND logits varied with time from 10 days to one year after stroke. The correlations were low or very low at the subacute phase, but moderate and strong at the chronic stage after stroke. At 10 days and 4 weeks, correlation of ABILHAND logits with movement time (−0.37, −0.32) and number of velocity peaks (−0.45, −0.30) were low, although correlations with mean and peak velocity remained very low (<0.13). At 3 months, all kinematic variables showed low correlation (<0.46) with ABILHAND logits. At 6 months, number of velocity peaks showed moderate correlation (−0.54), movement time and mean velocity showed low correlation (<0.49) and peak velocity showed very low correlation (0.15) with ABILHAND logits. At 12 months, movement time showed high correlation (−0.75), mean velocity and number of velocity peaks showed moderate correlation (<0.66) while peak velocity showed low correlation (0.36). Figure 3 shows the correlation coefficients between ABILHAND logits and kinematic variables between day 10 and month 12 after stroke.

## Discussion

This study examined the relationships between objective end-point kinematics obtained from the target-to-target pointing task and the self-reported manual ability in individuals with stroke during the first year after stroke. The results showed that correlations vary with time, and are low or very low early after stroke and became moderate to high after 6 months for objective measures of movement time and smoothness, but remained low to moderate for mean velocity and low for peak velocity.

The relationships between self-reported manual ability and objective kinematic measures were the weakest early after stroke. This result is not surprising since it is likely that patients in the early stage might have not had enough time to fully perceive how their stroke induced impairments influence their manual ability in daily tasks. The results from the current study showed that correlations between self-reported manual ability and kinematic measures, in general, were stronger at later time points after the stroke onset. This finding implies that, over time, the self-perceived manual ability is becoming closer to the actual manual performance measured with objective measures. This was particularly evident for two kinematic variables, movement time and number of velocity peaks. A longer movement time in the target-to-target task means that the task takes longer time to accomplish and an increased number of velocity peaks is indicative that movements are less smooth, that a movement was divided into several movement units and that the precision of movements was decreased. One possible reason for the observed stronger correlations of movement time and number of velocity peaks with ABILHAND logits at later time points might be that these deficits are more easily noticed in real life activities than others. Movement time and number of movement units are also strongly correlated (0.87) which might as well explain the similar pattern seen in these variables^21^. The relationship between ABILHAND logits and kinematic variables at later time points might have been influenced by the decreased variability when more participants reached near-maximal scores of the scales. This decrease in variability occurs, however, in both scales, and therefore have less impact on correlation coefficients. In this study, we also used Spearman’s correlation coefficient, since it is more robust to outliers and non-linear distribution that Pearson’s correlation coefficient.

Several past studies have explored the cross-sectional relationship between self-perceived and objectively assessed upper limb function, although they use different measurement scales. In the subacute stage of stroke, low correlation (0.3–0.4) was reported between objective kinematic measures from the drinking task and ABILHAND logits, similar to the results from the current study^14^. This indicates that the correlation of ABILHAND logits with kinematic variables are similar for both the drinking task and the pointing task. Another study in the subacute stage showed, however, high (0.8) correlation between grip strength measured using hand dynamometer and self-rated strength of the arm and handgrip reported by Stroke Impact Scale (SIS). The results from these studies imply that objective and self-reported measures might be more strongly coupled when the measured construct is specific (hand strength) compared to a wider construct as manual ability measured with ABILHAND in daily activities. ABILHAND assesses bimanual ability, while the kinematic measurements in the target-to-target or drinking task were performed unimanually, which might also have influenced the relationship between these assessments. In the chronic stage of stroke, moderate to high correlations (0.61–0.91) were found between self-perceived amount of arm use and Fugl-Meyer Assessment of Upper Extremity^2,6^. These results are in line with the findings from the current study, demonstrating stronger correlations at the chronic stage of stroke.

The discrepancy between self-perceived and observed upper limb function among well-performing individuals with stroke has been documented in literature. Approximately 20–40% of individuals with perfect or near perfect arm function scores in the observational scales reported residual disability in the self-perceived assessment scales^7,9^ during the subacute and chronic stages after stroke. As high as 75% individuals reported reduced amount of arm use in the subacute stage of stroke, even though they had near-perfect scores in an activity capacity scale^8^. Results from an upper limb training trial showed that the increase in arm function over time did not correspond to the increase in self-perceived arm function in 23% individuals at the subacute stage of stroke^3^. The discrepancy between self-perceived and objectively measured manual ability was also evident in the current study and particularly in the subacute stage of stroke.

The main barrier during goal-setting in stroke rehabilitation is the difference in clinicians’ and patients’ perspective of goal setting^22^. The goals suggested by clinicians are likely to be based on the objective assessments of arm function, but the goals important to the patients will more likely be related to their perceived function. It is evident from the current study that self-perceived manual ability might be weakly correlated to the objective assessments in the acute and subacute stage, and that this correlation might become stronger in chronic stages after stroke. Therefore, a combination of self-reported and objective assessments of upper limb is needed for individualized goal setting after stroke.

The strength of this study is that a relatively large, unselected cohort was followed from as early as within 10 days up to 12 months after stroke, allowing for understanding the variation of correlation between self-reported and objective measurements. The assessments were performed in a timely manner at all stages after stroke as suggested in the framework of Stroke Recovery and Rehabilitation Roundtable^23^. The results of this study can be generalized to post-stroke individuals with moderate to mild stroke impairment of the upper limb.

This study is not without limitations. The symptoms and sequelae associated with stroke, such as apraxia, post-stroke depression, decreased emotional control, and other cognitive impairments might lead to reduced ability to properly report the self-perceived manual ability. Unless severe, these possible deficits were not considered in this study. The relationship between ABILHAND logits and kinematic variables might have been influenced by the decreased variability when more participants reached near maximal scores of the scales. This decrease in variability occurs, however, in both the scales and therefore have less impact on the correlation coefficients. In this study, we also used the Spearman’s correlation coefficient, since it is more robust to outliers and non-linear distributions than the Pearson’s coefficient^24^.

To conclude, this study shows that the relationship between self-perceived manual ability and objective assessment of arm function varies with time in individuals after stroke. The discrepancy between self-perceived and objective assessment of arm function is more prominent in the early stages after stroke. Therefore, combination of self-reported and objective assessments of upper limb is needed to better understand the individual’s perspective after stroke, especially in the acute and subacute stages after stroke.

## Data Availability

The dataset used and/or analyzed during the current study is not publicly available because further data analysis is ongoing. But the dataset is available from the principal investigator, Katharina S. Sunnerhagen (ks.sunnerhagen@neuro.gu.se) on reasonable request.
